# Expression and Localization of Fas-Associated Factor 1 in Testicular Tissues of Different Ages and Ovaries at Different Reproductive Cycle Phases of *Bos grunniens*

**DOI:** 10.3390/ani13030340

**Published:** 2023-01-18

**Authors:** Jingyu Wang, Yangyang Pan, Rui Zhang, Gengquan Xu, Rentaodi Wu, Wenlan Zhang, Xiaoshan Wang, Xue Su, Qintuya Si, Sijiu Yu

**Affiliations:** 1 College of Veterinary Medicine, Gansu Agricultural University, Lanzhou 730070, China; 2 Alxa League Animal Husbandry Research Institute, Alxa 750300, China

**Keywords:** *Bos grunniens*, Fas-associated factor 1, expression localization, reproductive cycle, specific primer design, testicular tissues, ovaries, real-time quantification

## Abstract

**Simple Summary:**

In order to explore the biological role of Fas-associated factor 1 (FAF1) in testes and ovaries of domestic yaks, we examined the mRNA, protein expression and tissue localization of FAF1 in the ovaries of different ages and reproductive cycles in domestic yaks. The results showed that FAF1 mRNA and protein were expressed differently in the ovaries of testes of different ages and different reproductive cycles, mainly expressed in sperm, sperm cells, spermatogonia, primary spermatoblasts, supporting cells, testesian stromal cells and periductalcells in the testes, germinal epithelial cells, granule cells, cumulus cells, follicular membrane cells and luteal cells of ovarian tissues, and it was speculated that FAF1 may be closely related to the testicular development, spermatogenesis and testosterone secretion of male domestic yaks, and to the follicular atresia of female domestic yaks. Development, maturation and luteal lysis physiological processes are related, which provides a basis for further exploration of FAF1 in the reproductive physiological function of domestic yaks.

**Abstract:**

Fas-associated factor 1 (FAF1), a member of the Fas family, is involved in biological processes such as apoptosis, inflammation, cell proliferation and proteostasis. This study aimed to explore the biological role of FAF1 in testicular tissue at different ages (juveniles (1 and 2 years old), adults (3, 4, 6, and 7 years old) and old-aged animals (11 years old)) and ovaries during different reproductive cycle phases (follicular, luteal, and pregnancy phases). FAF1 mRNA, relative protein expression and protein expression localization were determined in testes and ovaries using real-time quantification, WB and immunohistochemistry (IHC), respectively. Real-time quantification of testis tissues showed that the relative expression of *FAF1* mRNA in testis tissues at 3, 4 and 7 years of age was significantly higher than of those in other ages, and in ovarian tissues was significantly higher in luteal phase ovaries than those in follicular and pregnancy phase ovaries; follicular phase ovaries were the lowest. WB of testis tissues showed that the relative protein expression of FAF1 protein was significantly higher at 11 and 7 years of age; in ovarian tissue, the relative protein expression of FAF1 protein was significantly higher in follicular phase ovaries than in luteal and pregnancy phase ovaries, and lowest in luteal phase ovaries. The relative protein expression of FAF1 at 3, 4 and 7 years of age was the lowest. IHC showed that FAF1 was mainly expressed in spermatozoa, spermatocytes, spermatogonia and supporting cells; in ovarian tissue, FAF1 was expressed in ovarian germ epithelial cells, granulosa cells, cumulus cells and luteal cells. The IHC results showed that FAF1 mRNA and protein were significantly differentially expressed in testes of different ages and ovarian tissues of different reproductive cycle phases, revealing the significance of FAF1 in the regulation of male and female *B. grunniens* reproductive physiology. Furthermore, our results provide a basis for the further exploration of FAF1 in the reproductive physiology of *B. grunniens*.

## 1. Introduction

The FAF1 protein is a component of the death-inducing signaling complex (DISC). It is an evolutionarily conserved protein consisting of 650 amino acids [1], with a size of approximately 74 kDa, and contains several functional, structural domains, including ubiquitin-associating (UBA), ubiquitin regulatory X (UBX), and death effector domains [2]. FAF1 mRNA and protein are expressed in human and mouse testes and ovaries [3]. The mouse *FAF1* gene is mapped to the chromosome 4C6 region. Northern blott analysis showed that mouse *FAF1* mRNA was expressed in muscle, thymus, lung, liver, kidney, heart, brain, ovary and testicles, and the expression level in testes was higher. In addition, the *FAF1* gene is highly expressed in the brain of the late stage of mouse embryonic development. Western blot analysis showed that it was expressed in a variety of tissues, including the pancreas, thymus, spleen, liver, kidney, ovaries, brain and testes [3]. Human *FAF1* gene is mapped to the chromosome 1q32 region; *FAF1* mRNA is expressed in testes, bone muscles, heart, ovaries, prostate, thymus, colon, small intestine, eyelid, pancreas, kidney, liver, lung, brain and other tissues, among which testes, skeletal muscle and heart expression is higher [4]. However, the biological role of FAF1 in Bos grunniens testes and ovaries is still unknown.

*B. grunniens*, also known as banner cattle, is one of the original cattle species unique to the Qinghai–Tibet Plateau, generally distributed on the plateau at an altitude of 3000 to 5000 m. In addition, it provides meat, milk, wool, leather, fuel, and other production and living materials for local herdsmen and is known as the “treasure of herdsmen” in Tibetan areas. Moreover, because of its unique living environment, it is referred to as “the boat of the plateau” and “the car of the glacier [5]”. Despite its importance, the reproductive rate of *B. grunniens* is low [6], a characteristic related to many factors, including sperm quality and ovarian function. High-quality semen is essential to ensure fertilization and early embryonic development, and good ovarian function is essential for fertilization [7]. Apoptosis is one of the mechanisms of reproductive regulation, and the cyclic changes in germ cells (sperm and oocytes) are closely related to their apoptotic processes. The Fas-FasL system is considered a critical physiological regulator of germ cell (sperm and egg) apoptosis [8]. FAF1 is an essential regulator of the Fas-FasL apoptotic pathway, acting on apoptosis in multiple ways [9], with its expression high in the germline. Therefore, it is essential to study the expression of FAF1 in testes of different ages and ovaries of different reproductive cycle phases. In this study, we quantified the relative expression levels of *FAF1* mRNA in testis tissues at different ages and ovaries at different reproductive cycle phases of *B. grunniens* using real-time. Furthermore, we employed western blotting to determine the relative FAF1 protein expression levels of the samples and immunohistochemistry to determine FAF1 protein localization. Our findings will provide a basis for further studies on the function of FAF1 in reproduction of *B. grunniens*.

## 2. Materials and Methods

### 2.1. Main Instruments and Reagents

The main instruments and equipment used in this pilot study included a Mastercycler X50 PCR instrument (Eppendorf, Framingham, MA, USA), LightCycler 96 real-time quantitative PCR instrument (Roche, Basel, Switzerland), DP71 microscope (Olympus Corporation, Tokyo, Japan), thermostatic incubator (Panasonic, Osaka, Japan) and an Amersham Imager 600 multifunctional imager (GE HealthCare, Chicago, IL, USA). The primary reagents used in this experiment included TransZol, RIPA lysis solution (TransGen Biotechnology Co., Ltd., Beijing, China), Taq enzyme (TaKaRa, Dalian, China) and goat anti-rabbit IgG-HRP, SP kit (Bioss, Beijing, China); reagents involved in WB experiments included (Solarbio Biotechnology Co., Ltd., Beijing, China), reverse transcription and SYBR^®^ Green Pro Taq HS premixed qPCR kit (Accurate Biology; Changsha, China). FAF1 rabbit anti-*B. grunniens* polyclonal antibody was prepared and preserved by Gansu Provincial Cattle and Sheep Embryo Engineering Center (Lanzhou, China).

### 2.2. Sample Collection

The test samples were collected in October 2021 at a slaughterhouse in Xining, Qinghai Province. A total of 21 healthy male *B. grunniens* of different ages and 9 healthy female *B. grunniens* of similar age and in different reproductive cycle phases (follicular stage ovaries with mature follicles, luteal stage ovaries with visible corpus luteum and pregnancy stage ovaries with developing fetuses in the uterus) were selected. Testicular and ovarian tissues were rapidly collected after lethal bleeding via the carotid artery and rinsed thrice with 0.9% saline. Thereafter, the tissues were trimmed into blocks of approximately 1 cm^3^ and fixed in a 4% neutral paraformaldehyde solution for later immunohistochemical testing. The remaining tissues were wrapped in tin foil, placed in a lyophilization bag, then into a liquid nitrogen tank as fast as possible to prevent tissue degradation and deterioration and transferred to an ultra-low temperature refrigerator at −80 °C for subsequent molecular assays.

### 2.3. Extraction of Total Tissue RNA and Protein

Total RNA was extracted from the testis tissues of yaks at different ages and ovaries at different reproductive cycle phases using TransZol reagent, according to the manufacturer’s instructions. The concentration of the obtained RNA was adjusted uniformly and then reverse transcribed into cDNA using a reverse transcription kit and stored at −20 °C. Next, the extracted proteins were mixed with 4× SDS loading buffer at a 3:1 ratio, denatured in a boiling water bath for 10 min, and immediately stored in an ice bath for 5 min at −20 °C.

### 2.4. Specific Primer Design

According to the mRNA coding region of *B. grunniens FAF1* published in GenBank, specific primers were designed in NCBI Primer-BLAST. *β-actin* (actin) was selected as the internal reference gene for the test, and Shanghai Sangong (Shanghai, China) synthesized all primers [10]. *FAF1-F1* and *FAF1-R1* were used for real-time quantitative PCR, and the primer sequences are shown in Table 1.

### 2.5. Relative Expression of FAF1 Gene in Testis Tissues of Different Ages and Ovarian Tissues of Different Reproductive Cycle Phases

Different tissue cDNA working solutions were prepared to a final concentration of 100 ng/μL. Next, 10 μL 2X SYBR^®^ Green Pro Taq HS Premix, 1 μL cDNA working solution for the different tissues, 0.4 μL each *FAF1-F1* and *FAF1-R1* (the concentrations of primers was 100 μmoL/L) and 8.2 μL RNase free water were added to make a 20-μL reaction solution. The cycling conditions were pre-denaturation at 95 °C for 300 s, denaturation at 95 °C for 10 s, annealing at 58 °C for 30 s and extension at 72 °C for 16 s. A total of 40 cycles were performed. Four replicate groups were set up for each template, and the relative expressions of *FAF1* mRNA in testis tissues of different ages and ovarian tissues of different reproductive cycle phases were calculated using the 2^−ΔΔCt^ method.

### 2.6. Relative Expression of FAF1 Protein in Testicular Tissues of Different Ages and Ovarian Tissues of Different Reproductive Cycle Phases

SDS-PAGE separation and concentration gels were prepared according to the 8 and 5% concentration gel preparation systems, respectively, after which the denatured protein samples were subjected to electrophoresis. After electrophoresis, the proteins were transferred onto 0.45-μm polyvinylidene fluoride membranes by wet transfer, and the residual transfer solution was washed off the membranes with phosphate buffered saline with Tween 20 (PBST) after the transfer was complete. The secondary antibody (goat anti-rabbit IgG-HRP) was incubated in a shaker for 40 min at 1:8000 dilution and washed thrice for 20 min with PBST. The working solution was prepared by mixing the ultrasensitive chemiluminescence A and B liquids at 1:1, after which it was added dropwise to the PVDF membrane and protected from light. Subsequently, the working solution was added onto the PVDF film and protected from light for 5 s. Chemiluminescence detection was performed using an Amersham Imager 600 System. Based on the imaging results, the relative expression of FAF1 protein was analysed using the target grey value/internal reference grey value.

### 2.7. Localization of FAF1 in Testicular Tissues of Different Ages and Ovarian Tissues of Different Reproductive Cycle Phases

Tissues fixed in 4% neutral paraformaldehyde were rinsed in tap water for 24 h, then dehydrated in gradient alcohol using wine benzene transparency, followed by wax immersion and paraffin embedding. Afterward, the tissues were cut into 4-μm continuous sections using a slicer. The slides were baked at 60 °C for 6 h on a film dryer, dewaxed and hydrated downstream, and antigen repairing was performed using citrate. Next, the slides were placed in 0.01 mol/L citrate, heated until boiling, then maintained at medium heat for 10 min. After cooling to room temperature, the slides were washed thrice with phosphate buffered saline (PBS) for 3 min each time. Blocking was performed by incubation at 37 °C for 10 min using a 3% H_2_O_2_ solution and PBS. Goat serum working solution (SPA kit A) was added at room temperature for 15 min, and the primary antibody (rabbit anti-*B. grunniens* FAF1 polyclonal antibody) of the test group was diluted at 1:400 and incubated overnight at 4 °C in a wet box, while the negative control group was incubated overnight using PBS as primary antibody and washed thrice with PBS for 3 min each time. In addition, the samples were incubated with biotin-labelled goat anti-rabbit IgG (SPA kit B) overnight at 37 °C and with horseradish-labeled streptavidin working solution (SPA kit C) at room temperature for 15 min and washed 3 times with PBS for 3 min each time. Thereafter, the slices were developed using DAB chromogenic solution, re-stained with haematoxylin, fractionated with alcohol in hydrochloric acid and kept in tap water for 10 min untilto blue. After dehydration and transparency, the films were sealed and photographed under a microscope.

### 2.8. Data Analysis

One-way ANOVA was performed on FAF1 mRNA and protein relative expression using SPSS 25.0 (SPSS Inc., Chicago, IL, USA) with 3 replicates in each group. The post hoc test performed after ANOVA was LSD. The results are expressed as mean ± standard error. Graphpad Prism 8 (GraphPad Software, San Diego, CA, USA) was used for plotting. A *p* value of <0.05 is considered significant difference.

## 3. Results

### 3.1. Relative Expression of FAF1 mRNA in Testes Tissues of Different Ages

**Figure 1:**
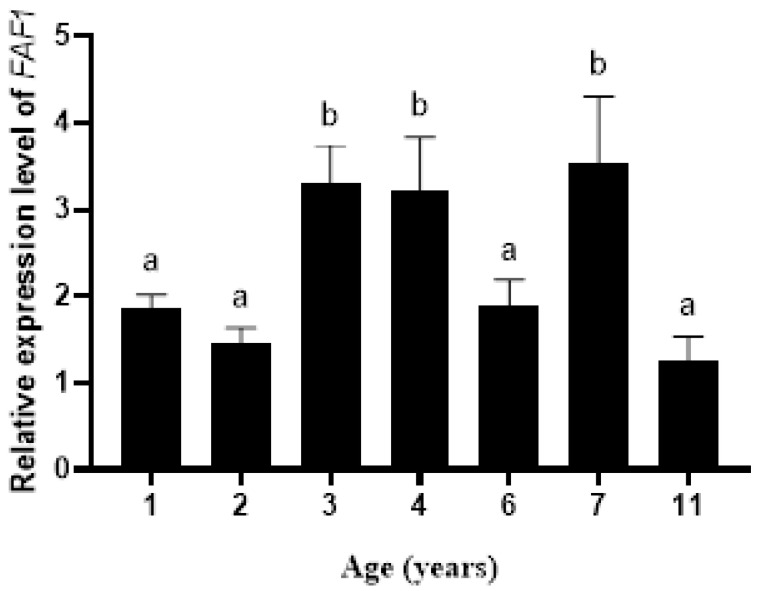
Relative expression levels of the *FAF1* gene in testis tissues of different ages (different letters indicate significantly different values at *p* < 0.05).

The qRT-PCR results (Figure 1) showed that *FAF1* gene expression was prevalent in testis tissues at different ages. The relative expression of the *FAF1* gene was significantly higher in testicular tissues at 3, 4 and 7 years of age than at other ages. In addition, the relative expression levels of the *FAF1* gene were differentially expressed in the testicular tissues of *B. grunniens* at different ages.

### 3.2. Relative Expression of FAF1 Protein in Testicular Tissues at Different Ages

**Figure 2:**
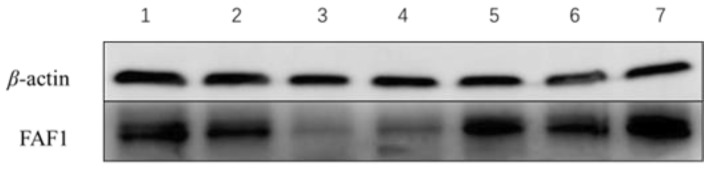
β-actin and FAF1 protein in testes of different ages.

**Figure 3:**
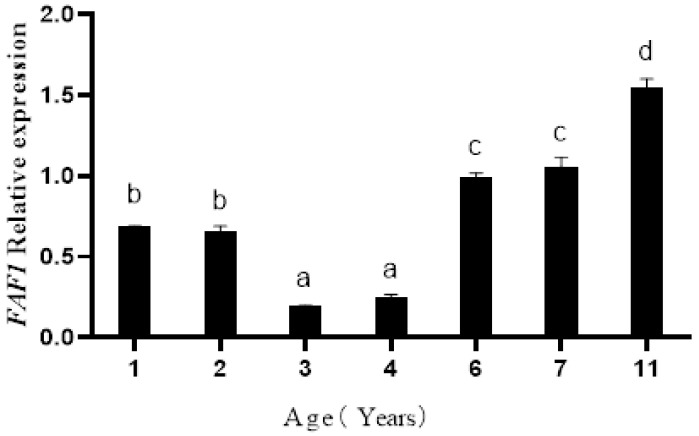
Relative expression levels of FAF1 protein in testis at different ages (different letters indicate significantly different values at *p* < 0.05).

Western blot results (Figure 2) showed that FAF1 protein was commonly present in testicular tissues of *B. grunniens* at different ages. The results (Figure 3) showed that the relative protein expression of FAF1 at 11 years of age was significantly higher than at other ages, followed by that at 6 and 7 years of age—the lowest relative protein expression of FAF1 was at 3 and 4 years of age. Therefore, the relative expression of FAF1 protein differed among *B. grunniens* at different ages.

### 3.3. Localization of FAF1 Protein Expression in Testicular Tissues of Different Ages

**Figure 4:**
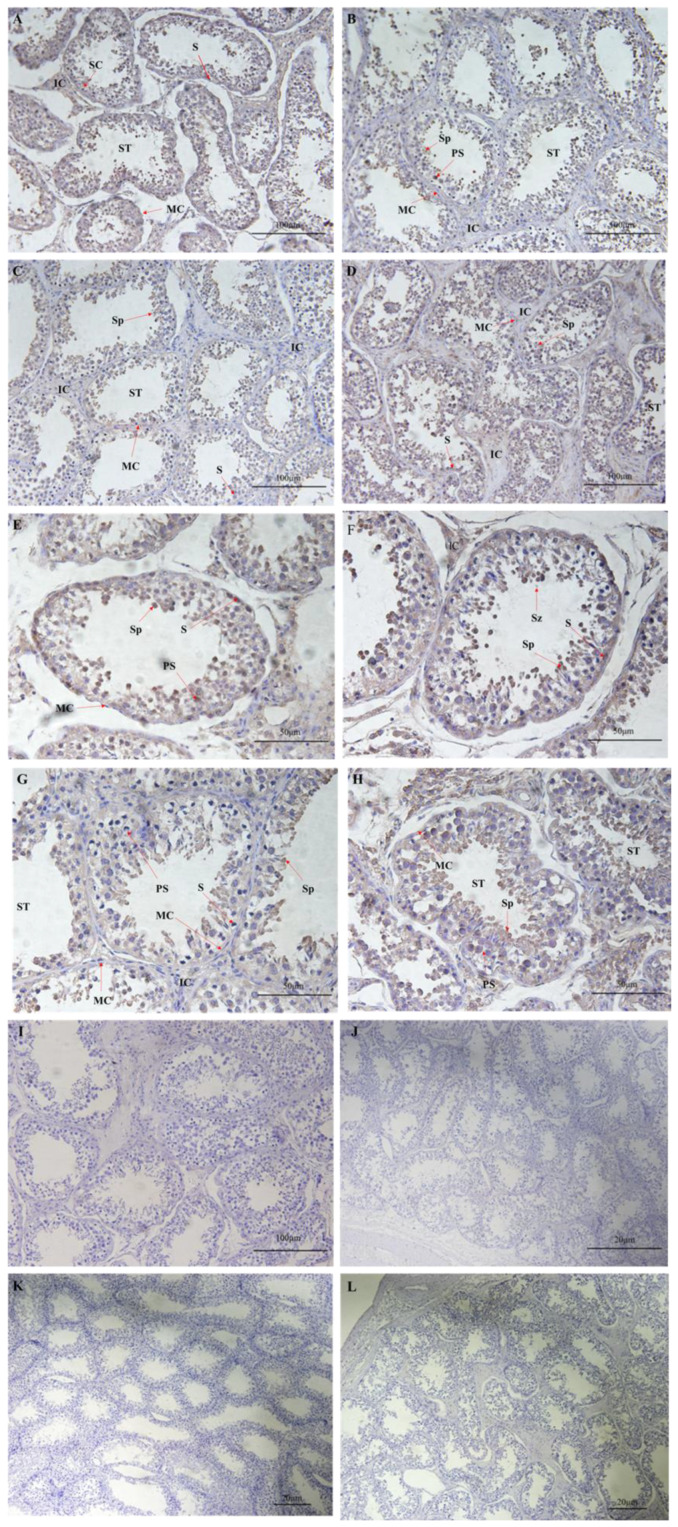
Localization of FAF1 protein expression in testes of different ages. (A–L) Immunohistochemical staining. (**A**–**H**) Positive expression of FAF1. (**I**–**L**) Negative control of FAF1. (**A**,**F**,**I**) One-year-old *B. grunniens* testis. (**B**,**F**,**G**) Four-year-old *B. grunniens* testis. (**C**,**G**,**K**) Seven-year-old *B. grunniens* testis. (**D**,**H**,**L**) Eleven-year-old *B. grunniens* testis. IC: interstitial; ST: seminiferous tubules; S: spermatogonia; PS: primary spermatocytes; Sp: sperm cell; SC: Sertoli cells; MC: peritubular myoid cells: Sz: spermatozoa.

The results of immunohistochemical staining (Figure 4) showed that FAF1 protein was positively expressed in the brown color in *B. grunniens* testes of different ages, and the distribution of FAF1 protein expression sites in testes of different ages was the same. FAF1 was mainly expressed in spermatozoa, spermatocytes, spermatogonia, primary spermatocytes, supporting cells, testicular interstitial cells, peritubular myeloid cells and germinal tubules.

### 3.4. Relative Expression Levels of FAF1 Genes in Ovaries of Different Reproductive Cycle Phases

**Figure 5:**
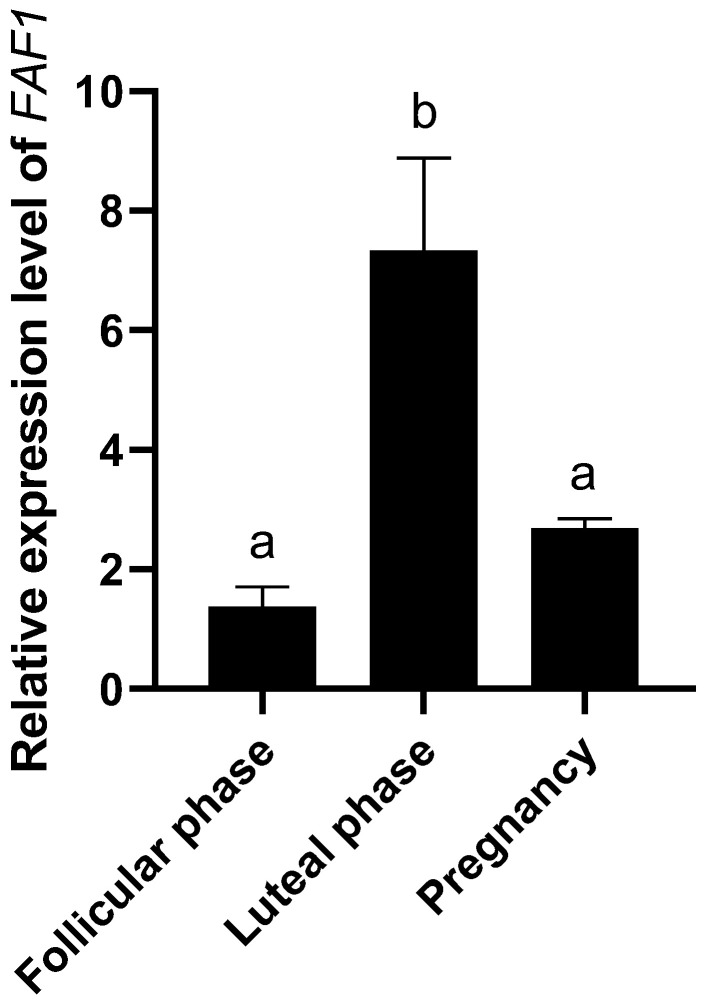
Relative expression of *FAF1* mRNA in the ovaries of different reproductive cycle phases in *B. grunniens* (different letters indicate significantly different values at *p* < 0.05).

The qRT-PCR results (Figure 5) showed that *FAF1* mRNA was commonly expressed in ovaries of different reproductive cycle phases. The relative expression of *FAF1* mRNA was significantly higher in luteal phase ovaries than that in follicular and pregnancy phases, followed by pregnancy phase ovaries. The lowest relative expression of *FAF1* mRNA was in follicular phase ovaries. The relative expression of *FAF1* mRNA was differentially expressed in ovarian tissues of different reproductive cycle phases in *B. grunniens*.

### 3.5. Relative Expression Levels of FAF1 Protein in Ovaries of Different Reproductive Cycle Phases

**Figure 6:**
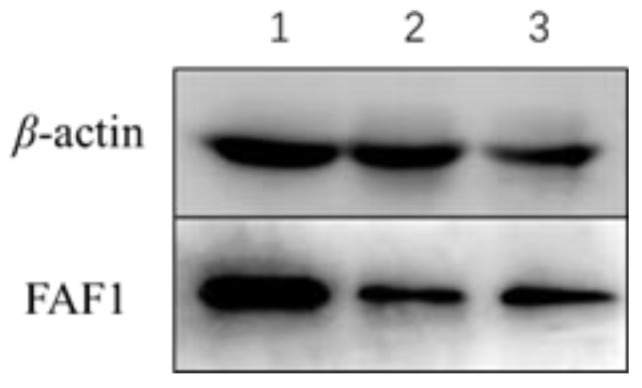
β-actin and FAF1 proteins detected in ovaries of different reproductive cycle phases. 1: Follicular ovary; 2: luteal phase ovary; 3: ovaries during pregnancy.

**Figure 7:**
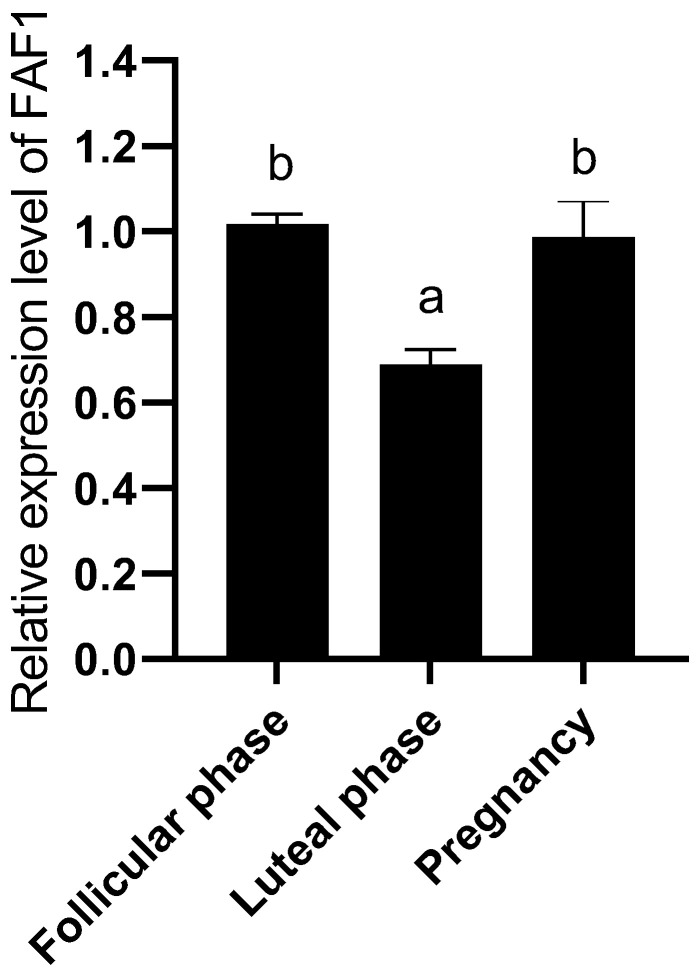
Relative expression levels of FAF1 protein in ovaries of different reproductive cycles (different letters indicate significantly different values at *p* < 0.05).

The Western blot results assay (Figure 6) showed that FAF1 protein was commonly present in ovarian tissues of different reproductive cycle phases of *B. grunniens*. The relative expression of FAF1 protein was significantly higher in follicular stage ovaries than in luteal and pregnancy stage ovaries, followed by pregnancy stage ovaries (Figure 7). The lowest relative expression of FAF1 was in luteal stage ovaries. Altogether, the results indicated that there was variability in the relative expression of FAF1 protein in the ovarian tissues of *B. grunniens* in different reproductive cycle phases.

### 3.6. Localization of FAF1 Protein Expression in Ovaries of Different Reproductive Cycle Phases

**Figure 8:**
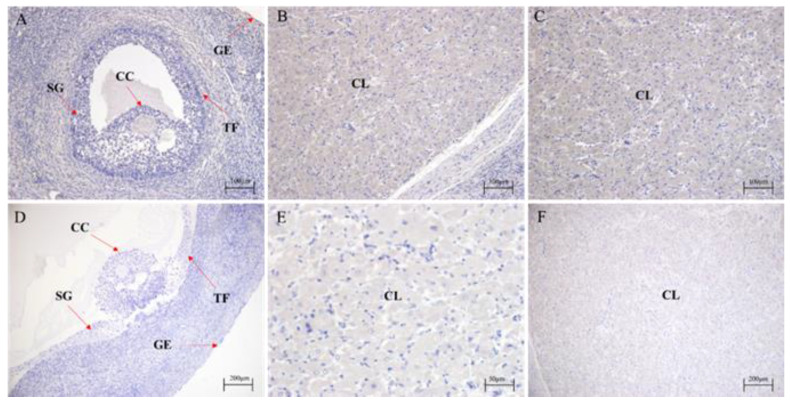
Localization of FAF1 protein expression in ovaries of different reproductive cycle phases. (**A**–**F**) Immunohistochemical staining. (**A**–**C**) Positive expression of FAF1. (**D**–**F**) Negative control of FAF1. (**A**,**D**) are follicular stage ovaries; (**B**,**E**) are luteal phase ovaries; (**C**,**F**) are ovaries during pregnancy; SG: granulosa cell; GE: germ epithelial cells; TF: follicle membrane cells; CL: luteal cells; CC: cumulus cell.

The immunohistochemical staining results (Figure 8) showed that FAF1 protein was positively expressed (in brown) in testes of *Bos grunniens* at different ages. The distribution sites were similar among ages, comprising spermatozoa, spermatocytes, spermatogonia, primary spermatocytes, supporting cells, testicular interstitial cells, peritubular myeloid cells and germinal tubules.

## 4. Discussion

FAF1 contains several functional and structural domains, primarily N-terminal UBA, C-terminal UBX, death effector domain interacting domain(DEDID) and FAS-interacting domain(FID). UBA can recruit polyubiquitinated proteins essential for FAF1-mediated apoptosis and stress responses [6,11], thereby mediating apoptosis and stress responses. UBX binds to the ubiquitin–proteasome system via a molecular chaperone-containing protein (valosin-containing protein, VCP) [11,12], the mammalian homolog of multifunctional Cdc48p in yeast and multifunctional p97 in *Xenopus laevis* [13], associated with various cellular activities. DEDID can be associated with Fas-associated protein with death domain (FADD) proteins and caspases [14]. FID interacts directly with Inhibitor of kappa B kinase β(IKKβ) to prevent the formation of IKKα, IKKβ and IKKγ/NEMO complexes, thereby inhibiting NF-κB signaling [15]. FAF1 is also known as an inhibitor of IκB kinase (IKK) activation [16]. In addition, FAF1 can also inhibit NF-κB activity by interfering with the nuclear translocation of the RelA subunit (p65) of NF-κB [17,18]. In recent years, FAF1-associated studies have also reported that FAF1 is an oncogene with reduced expression in various tumor tissues and cells [19], such as gastric [20,21], cervical [22] and breast cancer [23] and mesothelioma [5]. FAF1 expression downregulation may lead to tumorigenesis, and FAF1 also regulates the antiviral immune process by inhibiting Mitochondrial Antiviral Signaling Protein(MAVS).

The testis is the gonad of male animals and is the main organ for sperm formation and testosterone secretion [24]. Spermatogenesis in the mammalian testis is a complex and delicate physiological process with an apoptotic regulatory mechanism. Spontaneous germ cell apoptosis occurs during normal spermatogenesis [25,26], and this process excludes germ cells that have been damaged. The Fas signaling pathway is closely related to the sensitivity of germ cells and may determine male fertility. In addition, it is closely related to and may dominate germ cell apoptosis [27]. FAF1 is an essential regulatory element of the Fas pathway and has an important regulatory role in Fas pathway activation [9]. The present study showed that as *B. grunniens* age, *FAF1* mRNA and FAF1 protein are commonly expressed at all ages, from juvenile to young to old, but there is variability at different ages, which is consistent with the findings of Adham et al. in mouse testes [3]. The differential *FAF1* mRNA relative expression increased at 3 and 4 years of age, decreased at 6 years of age, increased again at 7 years of age and then decreased again, suggesting that FAF1 plays an essential biological role in physiological processes such as testicular development and spermatogenesis. *FAF1* mRNA relative expression increased as male *B. grunniens* reached sexual maturity, probably due to an increase in the number of germ cells as a result of altered testosterone secretion at sexual maturity. This results in increased apoptosis of testicular germ cells [28] and, thus, the relative expression of *FAF1* mRNA increases. The decreased FAF1 expression in old age may be due to reduced testosterone secretion, sexual function, spermatogenesis and apoptosis. The relative expression of FAF1 protein decreases in early youth (3 and 4 years old) and then gradually increases, unlike the relative expression of *FAF1* mRNA. This is probably because the FAF1 protein cannot initiate the apoptotic program alone, except in human BOSC23 cells, where it can directly initiate apoptosis [29]. FAF1 protein must bind to other proteins to form a death effector filament (DEF) or DISC to exert apoptotic effects [16]. FAF1 protein is mainly expressed in spermatozoa of different ages, spermatocytes, spermatogonia, primary spermatocytes, supporting cells, testicular mesenchymal cells, peritubular myeloid cells and germinal tubules. Furthermore, it supports cells, provides nutrition and supports germ cell proliferation and differentiation. Testicular interstitial cells secrete testosterone and influence the differentiation of support cells. Peritubular myoid cells mainly cooperate with support cells, provide substrate for spermatogonia and produce spermatogonia at different stages. Germinal tubules are the site of spermatogenesis [30], suggesting that FAF1 may be involved in testicular development, spermatogenesis, testosterone secretion and other critical physiological processes.

The ovary plays a crucial role in animal reproduction because it produces oocytes and secretes several sex hormones. The relative expression of *FAF1* mRNA is significantly higher in luteal phase ovaries than in follicular and pregnancy phases, followed by pregnancy phase ovaries, with the lowest being in follicular phase ovaries. In ovarian tissue, apoptosis occurs in atretic follicles and ovarian epithelium at ovulation and during luteal onset [31]. *FAF1* mRNA expression increases significantly in luteal phase ovaries due to the formation of the corpus luteum and increased progesterone secretion. Taniguchi et al. found that the level of *Fas* mRNA expression was significantly higher in bovine luteal phase ovaries than in other phases [32]. FAF1 is a member of the Fas family and is an essential regulatory progenitor of the Fas pathway. The relative expression of FAF1 protein was significantly higher in follicular stage ovaries than in luteal and pregnancy stages, followed by pregnancy stage ovaries. The lowest relative expression was in the luteal phase. FAF1 is expressed in the ovary’s germinal epithelium, granulosa cells, oocytes, and luteal cells in different reproductive cycle phases. The germinal epithelium is an essential factor in oocyte formation [33]. The proliferation and differentiation of granulosa cells are closely related to follicle development [34], and follicular atresia is essentially the result of granulosa cell apoptosis [35]. Oocytes regulate the expression of hormone proteins and related regulatory factors through gap junction expression, thereby affecting oocyte maturation [36]. Luteal cells are essential in pregnancy establishment and luteal maintenance, development and degeneration [37,38]. It is therefore hypothesized that FAF1 protein may be involved in follicular atresia, development, maturation and luteolytic physiological processes.

## 5. Conclusions

FAF1 was expressed in testes of different ages and ovaries of different reproductive cycle phases of *B. grunniens*. The expression levels of FAF1 were significantly different in testes of different ages and ovaries of different reproductive cycle phases, revealing that FAF1 has an essential biological role in the different life stages of *B. grunniens*. It is hypothesized that FAF1 may be closely related to testicular development, spermatogenesis and testosterone secretion in male *B. grunniens* and follicular atresia, development, maturation and luteolysis in female *B. grunniens*. However, the mechanisms involved need to be further investigated.

## Figures and Tables

**Table 1:** Primer sequences and lengths. T_m_—melting temperature.

Primer	Primer Sequence	Tm	PCR Product Lengths (bp)	GenBank Accession Number
*FAF1*-F1	5′-GGTGGATGATGGAGAAGTAT-3′	49.5	232	MK_416195.1
*FAF1*-R1	5′-GTGGAGGTAGATAGCAAGAA-3′	49.5		
*β-actin*-F	5′-CGTCCGTGACATCAAGGAGAAGC-3′	58	143	DQ838049.1
*β-actin*-R	5′-GGAACCGCTCATTGCCGATGG-3′	60		

## Data Availability

The datasets presented in this study can be found in online repositories. The names of the repository/repositories and accession number(s) can be found in the article/Supplementary Materials.

## Supplementary Materials

The following supporting information can be downloaded at: https://www.mdpi.com/article/10.3390/ani13030340/s1, Figure S1: β-actin protein in testes of different ages; Figure S2: FAF1 protein in testes of different ages; Figure S3: β-actin proteins detected in ovaries of different reproductive cycle phases; Figure S4: FAF1 proteins detected in ovaries of different reproductive cycle phases; Table S1: qRT-PCR raw data; Table S2: Testicular tissue of WB data of different ages; Table S3: Reproductive cycle ovarian tissue of WB data.
