# Supplementary figures and images for: Expression and Localization of Fas-Associated Factor 1 in Testicular Tissues of Different Ages and Ovaries at Different Reproductive Cycle Phases of Bos grunniens
Source: Animals (Basel). 2023 Jan 18;13(3):340. doi: 10.3390/ani13030340 (PMC9913830; doi:10.3390/ani13030340)

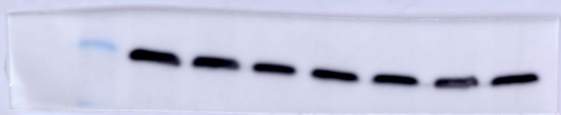

Supplement: Supplementary file 1 [file animals-13-00340-s001.zip › Figure S1. Beta-actin protein in testes of different ages.pdf]

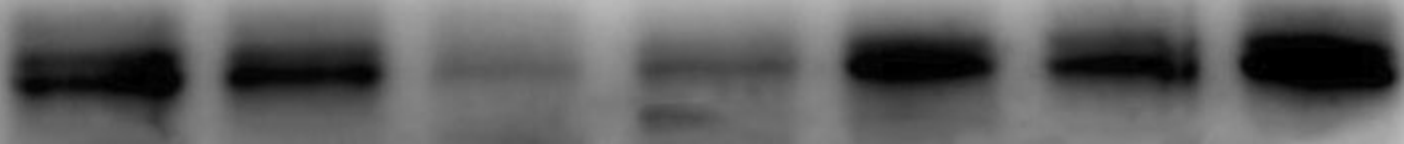

Supplement: Supplementary file 1 [file animals-13-00340-s001.zip › Figure S2. FAF1 protein in testes of different ages.pdf]

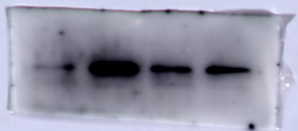

Supplement: Supplementary file 1 [file animals-13-00340-s001.zip › Figure S3. Beta-actin proteins detected in ovaries of different reproductive cycle phases (2).pdf]

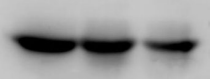

Supplement: Supplementary file 1 [file animals-13-00340-s001.zip › Figure S3. Beta-actin proteins detected in ovaries of different reproductive cycle phases.pdf]

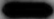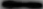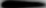

Supplement: Supplementary file 1 [file animals-13-00340-s001.zip › Figure S4. FAF1 proteins detected in ovaries of different reproductive cycle phases.pdf]
